# Editorial: Molecular Mechanisms of Proteinuria

**DOI:** 10.3389/fmed.2018.00300

**Published:** 2018-11-12

**Authors:** Sandra Merscher

**Affiliations:** Katz Family Division of Nephrology and Hypertension, Department of Medicine, Peggy and Harold Katz Family Drug Discovery Center, University of Miami Miller School of Medicine, Miami, FL, United States

**Keywords:** molecular, mechanisms and models, proteinuria, glomerular diseases, nephrology

Proteinuria is the hallmark of many glomerular diseases. Although major progress in elucidating the molecular mechanisms leading to the development of proteinuria has been made in the last two decades, it has become clear that these mechanisms are complex and involve many different pathways.

This research topic is a collection of review and mini review articles that highlights current advances in deciphering the molecular mechanisms leading to the development of proteinuria in glomerular diseases. Understanding these mechanisms may ultimately have major clinical implications and aid in identifying new targets and therapeutic strategies for patients affected by glomerular diseases.

The review article by Cil and Perwad summarizes the pathophysiology and genetics of nephrotic syndrome in children with a focus on monogenic causes. More than 50 genes which, when mutated, negatively affect the structural integrity and function of the glomerular filtration barrier thus contributing to the development of proteinuria, have been identified. Mutated genes include genes coding for slit diaphragm, actin cytoskeleton-related, mitochondrial, GBM-related proteins, nuclear transcription factors and proteins and proteins of the proximal tubule protein absorption pathways. The authors discuss the function of these genes in the context of nephrotic syndrome and conclude that genetic testing for mutations in these genes would offer a valuable opportunity for personalized treatment of patients with nephrotic syndrome in the future.

Focal segmental glomerulosclerosis (FSGS) is a histological lesion rather than a disease entity and an important cause of idiopathic nephrotic syndrome in children and adults. FSGS is characterized by variety of underlying etiologies ranging from genetic, metabolic and infectious to unknown origins. The article by Wen et al. reviews genetic causes of FSGS due to mutations in podocyte genes, the role of APOL1 in conferring susceptibility to FSGS in African American patients, the role of circulating permeability factors in the recurrence of proteinuria in patients with FSGS and the role of viral infections in disease pathogenesis. The authors conclude that FSGS is a complex disease with numerous divergent etiologies and pathogenic mechanisms which contribute to the variable response rate to therapy and recurrence post-transplantation and that there is a quest for reclassification of the disease in clinically relevant subtypes and for further exploration of relevant molecular pathways to develop novel therapeutic agents and therapies.

Another cause of nephrotic syndrome in children and adults is minimal change disease (MCD). Clinically and pathologically there is some overlap between MCD and FSGS suggesting that both diseases may have some shared molecular mechanisms leading to the development of proteinuria. However, if MCD evolves into FSGS over years remains the subject of controversy. In the review article by Bertelli et al., the authors discuss potential molecular triggers and mechanisms contributing to the development of proteinuria and the pathogenesis of MCD. The current controversy if MCD is an autonomous disease or if MCD and FSGS represent different manifestations of the same disease is also addressed. The authors describe the current knowledge coming from experimental studies in animal models suggesting the involvement of T-cell effectors, T-regulatory and B-cells in MCD. The role of cytokines, oxidants, B7-1(CD80), CD40/CD40L, c-Mip, TNF, uPA/suPAR, Angiopoietin-like 4 as potential candidate molecules implicated in the pathogenesis of MCD are also discussed. Whereas there seems to be some overlap in the molecules involved in the pathogenesis of MCD and FSGS, the authors propose that the pathogenesis of MCD is multifactorial and that MCD represents a “clinical-pathological-genetic entity” in which many molecules are involved.

HIV-associated nephropathy (HIVAN) is a form of secondary FSGS, characterized by severe proteinuria and progressive renal failure in the absence of treatment, occurring primarily in persons of African ancestry with advanced HIV disease. The review by Rednor and Ross discusses the roles of HIV genes, APOL1 gene variants, and molecular mechanisms contributing to glomerular and tubulointerstitial injury in HIVAN. They review research suggesting that HIV gene expression in podocytes contributes to cell cycle dysregulation, inflammation, cell death, and disturbances in the cytoskeletal homeostasis and that polymorphisms in the APOL1 gene may explain the increased susceptibility of patients of African descent to HIVAN. The authors stress that there is a need to determine whether podocytes may serve as a reservoir for HIV in HIV-positive patients treated with antiretroviral medications and to elucidate how HIV increases susceptibility in patients with APOL1 risk allele genotypes. Such insights may help to develop new therapeutic strategies for the prevention and treatment of HIVAN in HIV-positive patients and/or APOL1 nephropathies in HIV-negative patients.

Lupus nephritis (LN) is a common complication of systemic lupus erythematosus (SLE) and is associated with kidney damage and proteinuria. While the etiology of LN remains unclear, immune cell infiltration of the kidneys is frequently observed. Genetic variants of the human *ITGAM* gene encoding CD11b are strongly associated with susceptibility to SLE, LN, and other complications of SLE. In the mini review article by Khan et al., the authors discuss the role of CD11b in modulating pathways important in cell adhesion, migration, phagocytosis and Toll-like receptor signaling in the pathogenesis of LN. They then explore the possibility that pharmacological activation of CD11b by small molecules may proof beneficial in the treatment of LN by suppressing the systemic increase in inflammatory cytokines and reducing tissue accumulation of activated leukocytes and conclude that recent research offers novel mechanisms for development of therapeutics targeting CD11b for LN, SLE, and other autoimmune diseases.

Diabetic kidney disease (DKD) is defined as renal disease in the presence of diabetes. DKD is the single leading cause of end-stage renal disease in the United States. Podocytopenia, the loss of podocytes, and glomerular hypertrophy are hallmarks of progressive DKD, and the degree of podocyte loss correlates with severity of the disease. While the role of podocyte injury in DKD has been extensively studied, less is known about the role of glomerular endothelial cells and their contribution to the pathogenesis of DKD. In the mini review by Daehn, Daehn, the author discusses our current knowledge with regard to glomerular endothelial injury and the role of reactive oxygen species and mitochondrial stress in the pathogenesis of DKD. The author concludes that targeting elements of the cross-talk between glomerular endothelial cells and podocytes will provide novel avenues for therapeutic intervention in DKD.

## Author contributions

The author confirms being the sole contributor of this work and has approved it for publication.

### Conflict of interest statement

The author declares that the research was conducted in the absence of any commercial or financial relationships that could be construed as a potential conflict of interest.

